# Transcriptomic analysis reveals candidate genes associated with anther development in *Lilium* Oriental Hybrid ‘Siberia’

**DOI:** 10.3389/fpls.2023.1128911

**Published:** 2023-02-08

**Authors:** Tingting Dong, Lixuan Wang, Rui Wang, Xi Yang, Wenjie Jia, Mingfang Yi, Xiaofeng Zhou, Junna He

**Affiliations:** ^1^ Beijing Key Laboratory of Development and Quality Control of Ornamental Crops, College of Horticulture, China Agricultural University, Beijing, China; ^2^ Flower Research Institute, Yunnan Academy of Agriculture Sciences, Kunming, China

**Keywords:** *Lilium* spp., anther development, RNA-Seq, jasmonic acid, lignin, pectin

## Abstract

Lily (*Lilium* spp. and hybrids) is an important cut flower crop worldwide. Lily flowers have large anthers, which release a large amount of pollen that stains the tepals or clothing and thus can affect the commercial value of cut flowers. In this study, lily Oriental ‘Siberia’ was used to investigate the regulatory mechanism of lily anther development, which may provide information to prevent pollen pollution in the future. Based on the flower bud length, anther length and color, and anatomical observations, lily anther development was categorized into five stages: green (G), green-to-yellow 1 (GY1), green-to-yellow 2 (GY2), yellow (Y), and purple (P). Total RNA was extracted from the anthers at each stage for transcriptomic analysis. A total of 268.92-Gb clean reads were generated, and 81,287 unigenes were assembled and annotated. The number of differentially expressed genes (DEGs) and unique genes were largest for the pairwise comparison between the G and GY1 stages. The G and P samples were clustered separately, whereas the GY1, GY2, and Y samples were clustered together in scatter plots from a principal component analysis. Gene Ontology and Kyoto Encyclopedia of Genes and Genomes analyses of DEGs detected in the GY1, GY2, and Y stages revealed that the pectin catabolic process, hormone levels, and phenylpropanoid biosynthesis were enriched. The DEGs associated with jasmonic acid biosynthesis and signaling were highly expressed at the early stages (G and GY1), whereas the DEGs associated with phenylpropanoid biosynthesis were mainly expressed in the intermediate stages (GY1, GY2, and Y). The DEGs involved in the pectin catabolic process were expressed at advanced stages (Y and P). Cucumber mosaic virus-induced gene silencing of *LoMYB21* and *LoAMS* caused a strongly inhibited anther dehiscence phenotype, but without affecting the development of other floral organs. These results provide novel insights for understanding the regulatory mechanism of anther development in lily and other plants.

## Introduction

Lily is a popular cut flower that produces large anthers and abundant pollen (Tong et al., 2013; Sui et al., 2015; Sui et al., 2020). Following anther dehiscence, a large amount of pollen is released, which tends to result in staining of the tepals and reduces the commercial value of cut flower. The removal of the anthers before dehiscence reduces the aesthetic value of the flower and increases the labor costs. Therefore, effective control of anther dehiscence will improve the quality and value of cut lily flower while keeping the flower intact. Molecular research on anther development and dehiscence has gradually increased in the recent years and may enable the identification of candidate genes to prevent staining caused by lily pollen.

The anther comprises the pollen-containing sacs of the stamen and are composed of reproductive and sterile non-reproductive tissues. Anthers are responsible for the production and release of pollen to enable pollination and the sexual reproduction of plants. Anther development has been categorized into two simple stages in tobacco (*Nicotiana tabacum*) (Goldberg et al., 1993). The first stage involves anther morphogenesis, cell and tissue differentiation, and pollen mother cell meiosis; the second stage involves pollen grain differentiation, anther enlargement and filament elongation, tissue degeneration and dehiscence, and pollen release (Goldberg et al., 1993).

In the first stage, the anther wall differentiates into four layers, comprising the epidermis, endothecium, middle layer, and tapetum, from the outer layer to the inner layer (Sanders et al., 1999; Scott et al., 2004; Wilson et al., 2011). The epidermis protects the internal cells from external damage. The endothecium is critical for anther dehiscence and pollen release. The middle layer degrades during anther development, and delayed programmed cell death leads to male sterility in *Actinidia deliciosa* (Falasca et al., 2013). The tapetum provides nutrients and space for pollen development and release. Many genes play important roles in these processes in model plant species—for example, *DYSFUNCTIONAL TAPETUM 1* (*DYT1*) (Zhang et al., 2006), *ABORTED MICROSPORES* (*AMS*) (Sorensen et al., 2003; Xu et al., 2010), and *MALE STERILITY 1 1* (*MS1*) (Wilson et al., 2001; Ito et al., 2007; Yang et al., 2007a) are involved in the development and degradation of the tapetum, which is vital for pollen maturation.

In the second stage, as the pollen grains differentiate and mature, structural changes in the anther wall involve secondary thickening of the endothelial cell walls, which provides mechanical force for anther dehiscence (Nelson et al., 2012). Secondary wall thickening is usually achieved by deposition of lignin, and abnormal accumulation of lignin in the endothecium results in failure of anther dehiscence and pollen release (Thévenin et al., 2011). Studies on *nst1 nst2* and *myb26* mutants have shown that secondary wall thickening, specifically in the endothecium, is necessary for anther dehiscence (Mitsuda et al., 2005; Yang et al., 2007b; Yang et al., 2017). Polygalacturonase (PG) genes associated with degradation of pectic polysaccharides in the cell wall play an important role in anther dehiscence (Yang et al., 2013). *ARABIDOPSIS DEHISCENCE ZONE POLYGALACTURONASE 1* (*ADPG1*), *ADPG2*, and *QUARTET2* are PG genes that participate in anther dehiscence in *Arabidopsis* (Ogawa et al., 2009).

Certain plant hormones regulate anther development and pollen maturation, including auxin, gibberellins (GAs) and jasmonic acid (JA). Auxin and JA are important for stamen development, especially at the advanced developmental stages (Song et al., 2018). JA controls stamen development, and many genes associated with the JA biosynthesis and signaling pathway affect anther dehiscence (Stintzi and Browse, 2000; Ishiguro et al., 2001; Huang et al., 2017). Auxin plays an important role in coordinating anther dehiscence and pollen maturation and acts indirectly through JA (Cecchetti et al., 2008; Tabata et al., 2010; Song et al., 2013). Auxin negatively regulates lignin deposition in endothecium by inhibiting the expression of transcription factor MYB26 to control anther dehiscence (Cecchetti et al., 2013). An auxin maximum in the middle layer is crucial for correct stamen and pollen maturation in *Arabidopsis* (Cecchetti et al., 2017). Reduction of the auxin content at an advanced stage is necessary for anther dehiscence. Rice FT-INTERACTING PROTEIN 7 (OsFTIP7) affects the change in auxin content by suppressing the expression of *YUCCA 4* (*OsYUCCA4*) in the auxin synthesis pathway, thereby regulating anther dehiscence (Song et al., 2018).

Previous studies on the mechanism of anther development in lily have isolated and cloned several genes, which have been functionally analyzed. The transcription factors *LoAMS*, *LoMYB80*, and *LoMYB33* play important roles in anther development and the pollen maturation of lily (Sui et al., 2015; Sui et al., 2020; Liu et al., 2021). Lily PLASMA MEMBRANE INTRINSIC PROTEIN 2 (LoPIP2) is involved in desiccation induced anther dehiscence (Tong et al., 2013). Elucidation of the molecular mechanism of anther development in lily requires more intensive screening of genes. In the present study, the development of lily anthers was investigated by, first, observing the external morphology and anatomical characteristics of the anther; then, RNA-sequencing (RNA-seq) was performed. Important regulatory genes for anther development were screened, their expression levels were estimated, and two of the genes were silenced to explore their function. The results provide novel information for understanding the regulatory pathway of anther development in lily and identifying candidate genes with potential utility for molecular breeding of lily hybrids with non-dehiscent anthers.

## Methods

### Anther morphology and anatomical observation

Intact flower buds were sampled from lily Oriental Hybrid ‘Siberia’ plants grown at Gasa Town, Xinping County, Yuxi, Yunnan, China. The samples were collected at five developmental stages: green (G), green-to-yellow 1 (GY1), green-to-yellow 2 (GY2), yellow (Y), and purple (P). The lengths of the flower buds and anthers were measured using a Vernier caliper at each stage, and the anther color was recorded concurrently. The length of all anthers in a bud was measured using Image J software. The average length of the six anthers was taken as the anther length. Anthers at each developmental stage were embedded in paraffin sections were cut with a microtome. Anatomical cytological observation was performed with an optical microscope (Sui et al., 2015).

### RNA extraction, library construction, and sequencing

Total RNA was extracted from 15 anthers using the TIANGEN polysaccharide polyphenol RNA extraction kit in accordance with the manufacturer’s instructions. Three replicates were extracted at each developmental stage. The quality and quantity of the extracted RNA was detected using a NanoDrop 2000 spectrophotometer, Agilent 2100 bioanalyzer, and 1.0% agarose gel electrophoresis. A RNA library was constructed and sequenced with the assistance of the Beijing Nuohezhiyuan Bioinformatics Co., Ltd. (Beijing, China). Briefly, the sequencing procedure was as follows: for each sample, high-quality RNA was enriched using oligo d(T) beads. The enriched mRNA was disrupted into short fragments and reverse-transcribed into cDNA with random primers. The cDNA fragments were purified with the QIA Quick PCR purification kit, end-repaired, and ligated to Illumina sequencing adapters. The ligation products were selected according to their size by agarose gel electrophoresis and amplified by PCR. The PCR products were combined into a cDNA library and sequenced with an Illumina HiSeq 4000™ system.

### Sequence quality control and annotation

After sequencing, the raw reads were cleaned to remove adapter sequences, low-quality reads (Q value ≤20 or reads with a poly N >10%), and any contaminator sequences using the SeqPrep (https://github.com/jstjohn/SeqPrep) and Sickle (https://github.com/najoshi/sickle) tools. The high-quality sequencing reads were assembled using Trinity v2.15.0 to generate contigs and singletons.

To obtain comprehensive annotation information, all genes and transcripts were blasted against six public databases comprising the National Center for Biotechnology Information non-redundant protein (NR), Swiss-Prot, Protein Families (Pfam), Clusters of Orthologous Groups of proteins (COG), Gene Ontology (GO), and Kyoto Encyclopedia of Genes and Genomes (KEGG) databases. Transcription factors were analyzed and statistical information on transcription factor families were obtained using the Plant TFDB 4.0 database (http://planttfdb.cbi.pku.edu.cn/).

### Differential expression analysis and functional enrichment

To identify genes that were differentially expressed in comparisons between developmental stages, the gene expression levels were quantified as the number of transcripts per million reads (TPM) using RSEM (http://deweylab.github.io/RSEM/) with the default parameters. The DEGs were identified using the DESeq 2 software based on the following criteria: log_2_ (fold change) (>1 or <-1) and false discovery rate (*q*-value) <0.05.

GO analysis was performed to identify the DEGs that were significantly (*P*_-adj_ ≤ 0.05) enriched in GO terms. The GO enrichment analysis was conducted using Goatools (https://github.com/tanghaibao/Goatools).

KEGG pathway analysis was performed to identify the DEGs that were significantly (P_-adj ≤_0.05) enriched in metabolic pathways compared with the whole-transcriptome background. KEGG pathway analysis was conducted with KOBAS (http://kobas.cbi.pku.edu.cn/home.do).

### Quantitative real-time PCR validation

The cDNA library constructed from the total RNA extracts was used as the template for quantitative real-time PCR (qRT-PCR) analysis. The samples were analyzed with ABI Stepone™ Real Time System using SYBR Premix Ex Taq II (Takara, Dalian, China). The 20 μl reaction mixture included 10 μl SYBR Premix Ex Taq II, 1 μl (100 ng) template cDNA, and 0.5 μM of each PCR primer. The PCR thermal-cycling protocol was as follows: 95°C for 2 min, followed by 40 cycles at 95°C for 5 s, 60°C for 10 s, and 72°C for 30 s. The relative expression level of the target genes was calculated using the 2^-ΔΔ^ *^C^* ^t^ method. The *18S* rRNA gene was used as an internal control. All primers used are listed in **Supplementary Table S3**.

### Verification of the function of LoAMS and LoMYB21 by CMV silencing system

We used the cucumber mosaic virus (CMV) system to perform virus-induced gene silencing, which utilized the vectors CMV101, CMV201, and CMV301 (Tasaki et al., 2016; Wang et al., 2016). The vectors CMV201-LoAMS and CMV-LoMYB were constructed and transformed into *Agrobacterium tumefaciens* strain EHA105. The CMV101, CMV201, CMV201-LoAMS, CMV201-LoMYB21, and CMV301 *Agrobacterium* suspensions were cultured with shaking. Each bacterial culture was centrifuged and resuspended with the infection solution (10 mM MgCl_2_, 10 mM MES, and 100 μM acetosyringone) until OD_600_ = 0.5. The suspensions with the three CMV vectors were combined in equal volumes and allowed to stand for 3–5 h before the infection. Cut flowers with buds of about 6 cm were treated with the infection solution for 24 h, washed with water two times, and then cultured in a hydroponics system until the flowers opened. The anthers were observed, and the dehiscence phenotype was recorded, then photographed, and stored at –80°C for further analysis.

## Results

### Morphological and anatomical cytological characteristics of lily anthers

**Figure 1:**
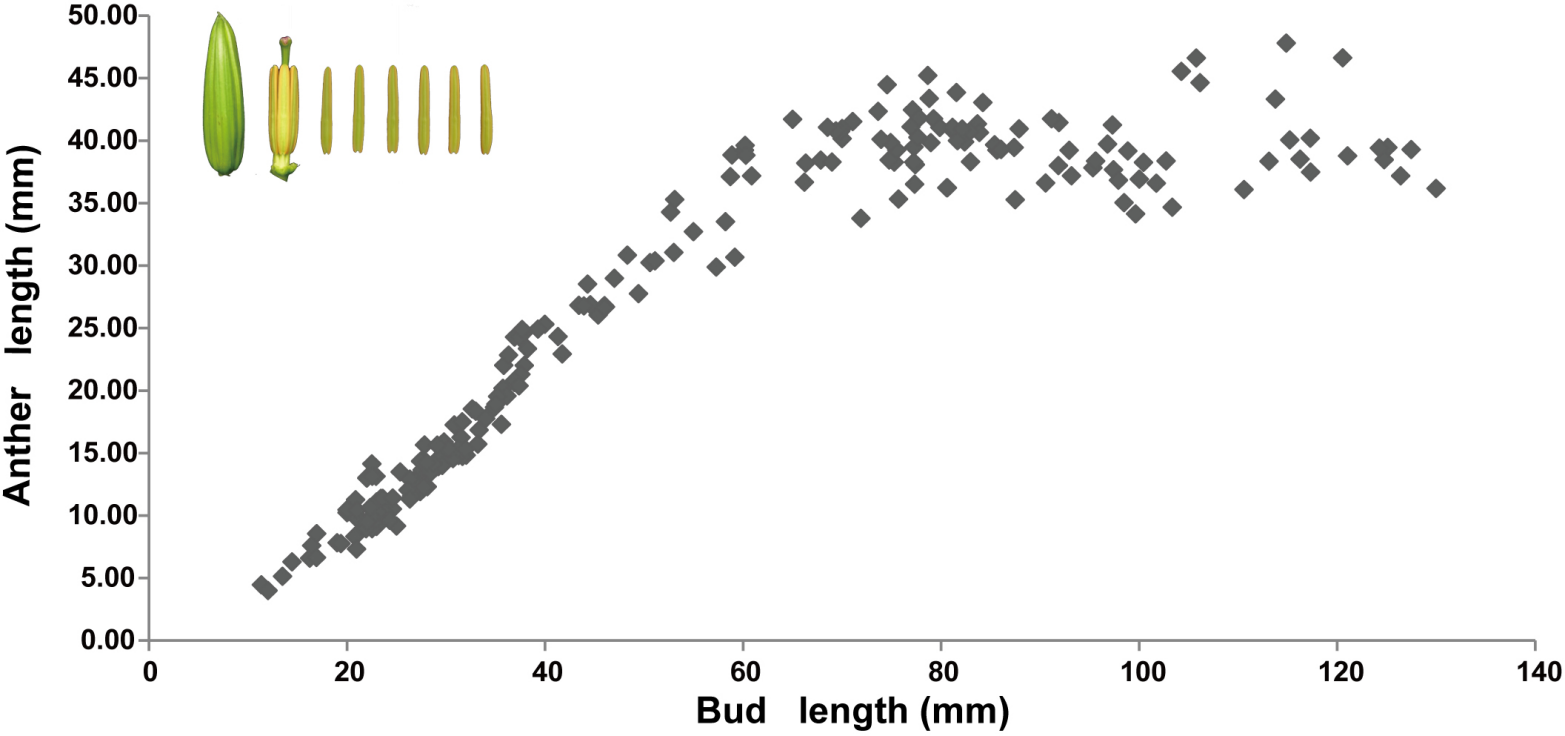
Relationship between anther length and flower bud length in *Lilium* Oriental Hybrid ‘Siberia’. In the upper left corner are images of the lily bud and anther. Each point represents the average length of six anthers. The abscissa is bud length, and the ordinate is anther length.

Anther development involves changes in external morphology, such as the anther length and color, as well as changes in the internal structure of the anthers. The relationship between the anther length and color and the bud length was examined. At a flower bud length of less than 6 cm, a linear relationship between anther length and bud length was observed, *i*.*e*., anther length significantly increased along with the increase in flower bud growth. If the bud length exceeded 6 cm, anther growth was extremely slow. For a bud length of more than 8 cm, the anther length remained stable and did not increase significantly with further bud development (**Figure 1**).

**Figure 2:**
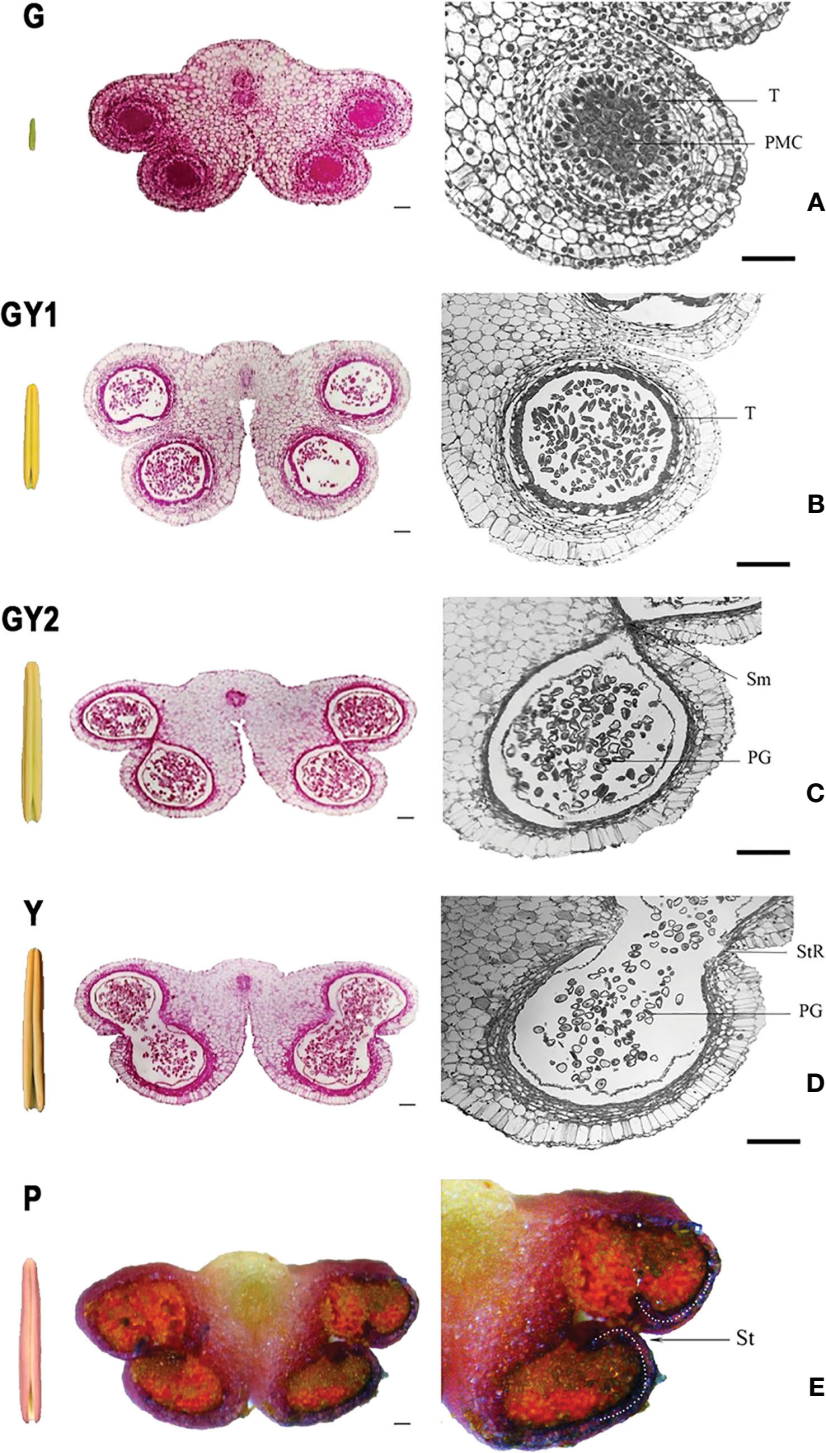
Anatomical structure of the anther at five developmental stages in *Lilium* Oriental Hybrid ‘Siberia’. Each stage includes a global external view of the anther, a transverse section of the complete anther with four locules, and a section of a single anther locule. **(A)** green stage **(G)**, **(B)** green-to-yellow 1 stage (GY1), **(C)** green-to-yellow 2 stage (GY2), **(D)** yellow stage (Y), and **(E)** purple stage (P). Bar = 100 μm.

The anther color changed during the growth process. The anthers were green in young flower buds less than 4 cm in length (**Figure 2A**). The anther color gradually changed to yellow with the flower bud development from 4 to 8 cm in length (**Figures 2B, C**). In flower buds longer than 8 cm, the anther color was yellow (**Figure 2D**). When the flower bud was longer than 10 cm, the color of the tepals epidermis gradually changed to white. The anther color changed from yellow to purple when the flower bud length was approximately 12 cm (**Figure 2E**).

The cellular structure of the anther also changed concurrently with the changes in length and color during anther development. The anther showed a typical “butterfly-shaped” transverse section, and the four anther wall layers were clearly observed in green anthers less than 4 cm in bud length, which was named as green stage (G) (**Figure 2A**). At the bud length between 4 and 8 cm and as the anther color changed from green to yellow, the cellular structure of the anthers differed between anthers that had just changed to yellow (GY1) and those that were completely yellow (GY2). During the GY1 stage, the bud length is between 4 and 5 cm, the color of the anther began to change to yellow with the side turning to yellow gradually and not uniformly, the anther developed pollen mother cells in the anther chamber, and the surrounding tapetum layer appeared, but no cell wall thickening in the anther chamber was observed. The microspores were released from the tetrad (**Figure 2B**). During the GY2 stage, the bud length is between 6 and 7 cm, and the color of the anther was kept the same as with GY1; the tapetum layer degenerated and was no longer closely connected with the endothecium (**Figure 2C**), which is different from the internal structure of GY1. In addition, the septum between the two anther chambers in the same anther sac began to dissolve, the tapetum cells had almost completely degraded, and secondary thickening of the endothelial cell wall was observed as banded deposition (**Figure 2C**). In a flower bud longer than 8 cm with yellow anther, the septum in each anther sac had completely degraded. The connection at the stomium was weak, and the anthers could begin to dehisce after exposure to air for several hours (**Figure 2D**). When the flower buds were ready to open, the exposed yellow anthers quickly turned purple and dehisced rapidly (**Figure 2E**).

### *De novo* assembly, quality assessment, and gene annotation of reads

To search for important genes that regulate anther development, especially anther dehiscence, the anthers were sampled at five developmental stages for RNA-seq. In total, 15 RNA libraries were constructed and sequenced. A total of 268.92 Gb clean data was obtained. The clean data per sample attained at least 14.24 Gb, and the Q30 value was more than 91.92% (**Supplementary Table S1**). Therefore, the quality of the obtained data was high, and further analysis was appropriate.

After data processing for quality control, a total of 81,287 unigenes and 117,458 transcripts were retained, and the N50 length was 1,791 bp (**Supplementary Table S2**). The transcript length distribution revealed that 31,075 sequences exceeded 1,000 bp, accounting for 38.23% of the total number of genes (**Supplementary Figure S1**). These results indicate that the data assembly is good and effective. Then, these data were used for function annotation. The unigenes obtained after assembly optimization were compared with six bioinformatics databases, and the corresponding functional annotation information was downloaded. Different proportions of unigenes were annotated in each database, *i*.*e*., 43.47% in NR, 37.50% in COG, 36.29% in GO, 30.62% in Swiss-Prot, 29.93% in Pfam, and 17.49% in KEGG (**Supplementary Figure S2**).

A BLAST search was conducted to match the unigenes with sequence data in the NR database. Overall, 17.92% of the unigenes had top hits to sequences from *Elaeis guineensis*, followed by *Phoenix dactylifera* (14.99%), *Asparagus officinalis* (7.71%), and *Ananas comosus* (5.34%) (**Supplementary Figure S3**). The transcription factor analysis showed that the highest proportion of the assembled unigenes were annotated as members of the MYB super family, followed by the AP2/ERF family and the bHLH family (**Supplementary Figure S4**).

**Figure 3:**
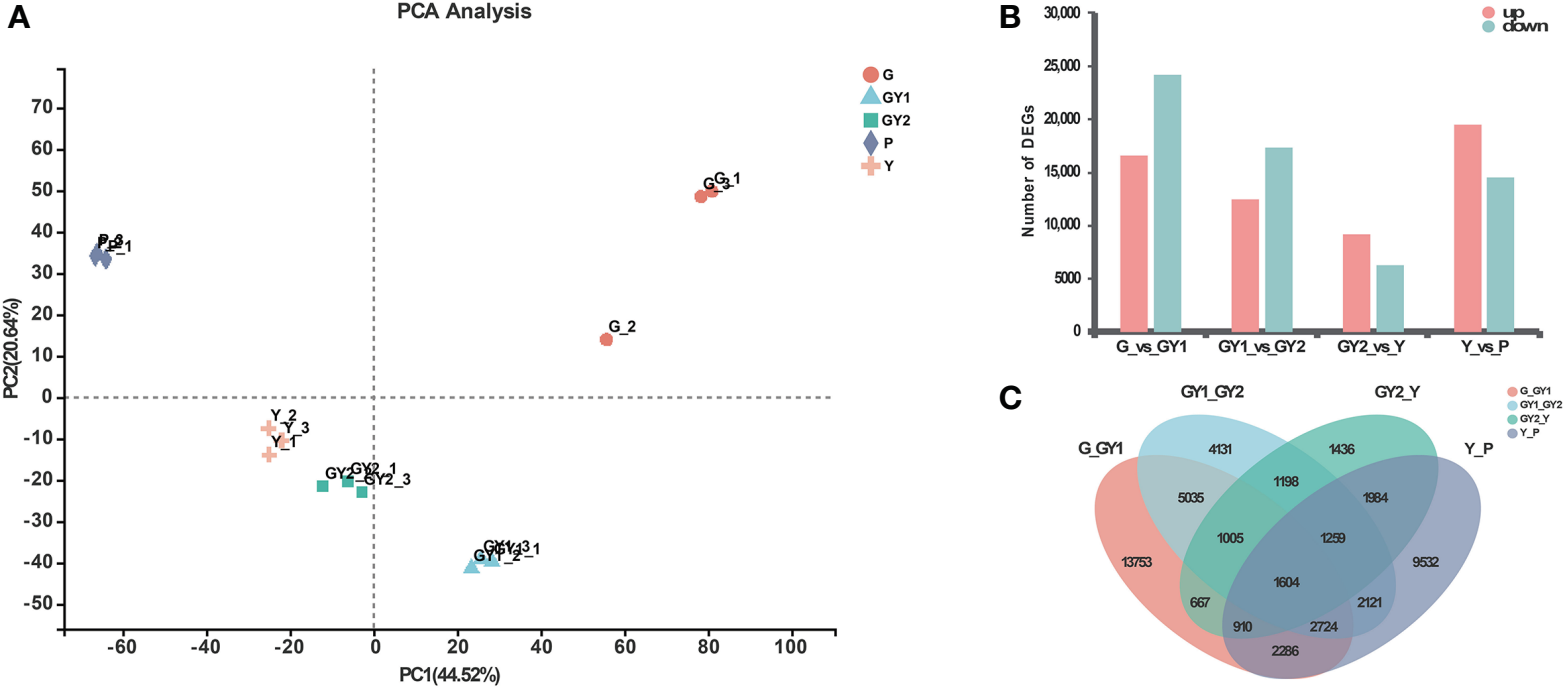
Differentially expressed genes (DEGs) at five stages of anther development in *Lilium* Oriental Hybrid ‘Siberia’. **(A)** Principal component analysis of different samples. The horizontal axis is the contribution of principal component 1, and the vertical axis represents the contribution of principal component 2. **(B)** Numbers of upregulated and downregulated DEGs in pairwise comparisons of five developmental stages: G-*vs*.-GY1, GY1-*vs*.-GY2, GY2-*vs*.-Y, and Y-*vs*.-P. **(C)** Venn diagram of the data presented in **(B)**.

The results of the principal component analysis (PCA) showed that the replicates of the RNA samples were clustered closely together in a scatter plot of the first and second principal components, and therefore the biological reproducibility of the samples was acceptable (**Figure 3A**). The samples for the G and P developmental stages were distinct, whereas the GY1, GY2, and Y samples were less well separated.

### Analysis of DEGs in all stages of anther development

To identify genes that were significantly differentially expressed between developmental stages, the expression levels of all genes were normalized to the TPM value. The DEGs among the libraries were identified by pairwise comparisons between the adjacent stages (**Figure 3B**). In the early stages of development, the highest number of DEGs was detected for the comparison G *vs*. GY1, comprising 40,916 genes, including 16,657 upregulated genes and 24,259 downregulated genes. In contrast, fewest DEGs were detected between GY2 and Y, consisting of 15,577 genes, including 9,246 upregulated genes and 6,331 downregulated genes (**Figure 3B**).

The number of DEGs in common among the four comparisons was visualized by generating a Venn diagram (**Figure 3C**). Among all DEGs, 1,604 genes were present in all four differential groups. Among the four differential groups, the highest number of unique genes was detected in the G *vs*. GY1 group (13,753 genes), which indicated that G and GY1 were the most developmentally distinct stages (**Figure 3C**).

Annotation of GO terms was performed to explore the potential functions enriched among the DEGs. The DEG functions were classified in three main components: biological processes, cellular components, and molecular functions. The top-ranked functions among annotated genes were binding (GO:0005488) and catalytic activity (GO:0003824) in the molecular function category, cell part (GO:0044464) and membrane part (GO:0044425) in the cellular component category, and cellular process (GO:0009987) and metabolic process (GO:0008152) in the biological process category. In addition, genes were annotated with transcription regulator activity (GO:0140110) and reproductive process (GO:0022414) (**Supplementary Figure S5A**).

The annotation of the DEGs with KEGG pathways showed that the 12,960 DEGs were mapped to 145 KEGG pathways. The three most/highly annotated pathways were metabolism of cofactors, vitamins, and carbohydrate metabolism in the metabolism category and translation in the genetic information category. In addition, genes were annotated with the signal transduction and cell growth and death pathways (**Supplementary Figure S5B**).

### Functional analysis of DEGs detected at the GY1, GY2, and Y developmental stages

**Figure 4:**
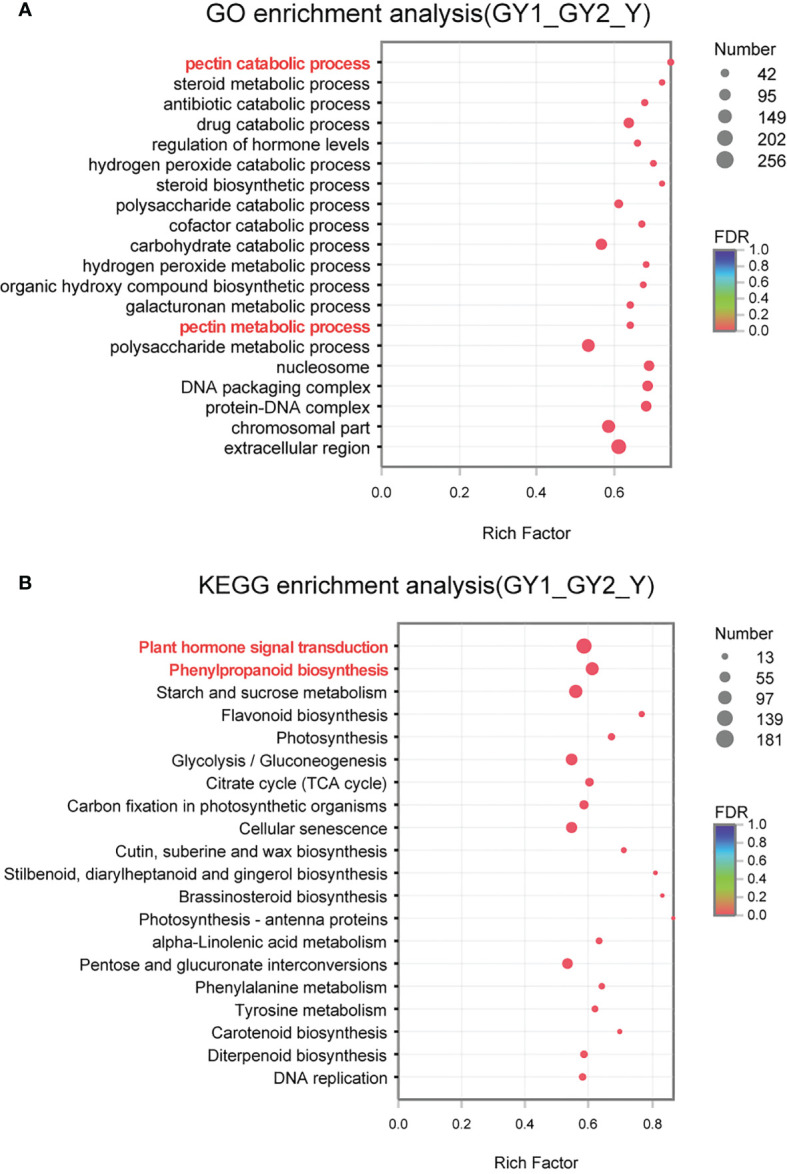
Gene Ontology (GO) and Kyoto Encyclopedia of Genes and Genomes (KEGG) enrichment analysis of differentially expressed genes (DEGs) detected in comparisons of the GY1, GY2, and Y stages of anther development. **(A)** GO enrichment analysis of DEGs. The vertical axis represents the GO term, and the horizontal axis represents the rich factor, the ratio of the number of enrichments to the number of annotations in the GO term. **(B)** KEGG enrichment analysis of DEGs. The vertical axis represents the pathway name, and the horizontal axis represents the rich factor, the ratio of the number of enrichments to the number of nodes in the pathway.

The PCA results showed that the RNA samples at the G and P stages were clustered separately, whereas the GY1, GY2, and Y samples were clustered together, indicating that the GY1, GY2, and Y stages were more strongly associated with each other (**Figure 3A**). The G stage is the initial phase of establishment of the anther wall structure, whereas the P stage corresponds to the final stage of anther dehiscence. The GY1, GY2, and Y stages involve diverse processes, such as secondary wall thickening, septum degradation, and stomium cell breakdown, which ultimately lead to anther dehiscence. Therefore, we focused on analyzing the DEGs detected in the GY1, GY2, and Y stages using GO and KEGG enrichment analysis (**Figure 4**).

GO enrichment analysis was performed to classify the potential functions of the DEGs involved in the GY1, GY2, and Y stages. The following functional groups were highly enriched: pectin catabolic process (56), steroid metabolic process (47), antibiotic catabolic process (59), drug catabolic process (127), regulation of hormone levels (59), hydrogen peroxide catabolic process (52), steroid biosynthetic process (42), and polysaccharide catabolic process (90) (**Figure 4A**). Pectin catabolic process and polysaccharide catabolic process are involved in cell wall metabolism, such as secondary thickening and degradation. This result was consistent with observations of the anatomical cytological characteristics (**Figure 2A**). The hydrogen peroxide catabolic process is associated with the activity of many enzymes, such as peroxidases, and reactive oxygen species, which play important roles in anther development. Many peroxidase genes were highly expressed at the GY1, GY2, and Y stages (**Supplementary Figure S6**), which indicated that reactive oxygen species play important roles in anther development.

Based on the annotations in the KEGG pathway database, the metabolic pathways significantly enriched among the three groups of DEGs were analyzed (**Figure 4B**), thus providing an improved understanding of the metabolic regulatory patterns involved in anther development. The highly enriched pathways were plant hormone signal transduction (181), phenylpropanoid biosynthesis (133), starch and sucrose metabolism (139), and flavonoid biosynthesis (30). Phenylpropanoid biosynthesis is associated with lignin deposition during secondary cell wall thickening. Starch and sucrose metabolism are associated with the energy used during pollen development.

Plant hormone pathways were clustered in both GO and KEGG enrichment analyses, and thus hormone pathways were important participants in anther development. In addition to JA and GAs, auxin, abscisic acid,and brassinosteroids play roles in this process. A total of 420 genes associated with plant hormones were detected, among which 146 genes were involved with auxin, followed by ethylene-related genes (134), and brassinosteroid-related genes were the fewest (7). The number of DEGs associated with auxin, ethylene, and GAs was the largest. By comparing the number of DEGs among the three groups, it was determined that several plant hormones showed similar characteristics. Most DEGs were detected in GY1 *vs*. GY2 groups, and the fewest DEGs were detected in the GY2 *vs*. Y group (**Supplementary Figure S7**).

### Expression of DEGs associated with JA synthesis and signaling pathways

**Figure 5:**
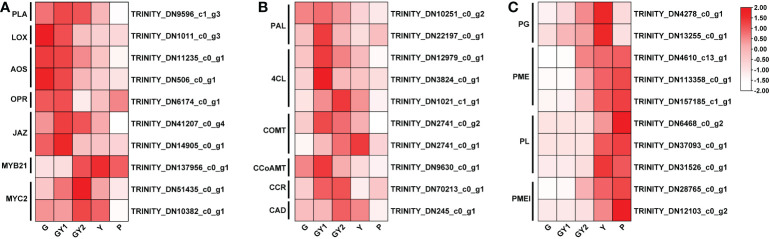
Differentially expressed genes (DEGs) involved in selected pathways associated with anther development. **(A)** Heat maps of the DEGs involved in jasmonic acid biosynthesis and signal transduction. **(B)** Heat maps of the DEGs associated with the lignin synthesis pathway. **(C)** Heat maps of the DEGs associated with pectinase activity. The red rectangles represent upregulated genes, and the white rectangles represent downregulated genes.

In the KEGG pathway enrichment analysis, plant hormone signal transduction formed the largest group (**Figure 4B**), indicating that hormones were important regulators of anther development. Previous research has shown that JA plays roles in anther development, filament elongation, and pollen viability (Cheng et al., 2009; Song et al., 2011; Qi et al., 2015). Therefore, genes involved in the JA synthesis and signaling pathways were analyzed. The JA synthesis genes *PHOSPHOLIPASE A* (*PLA*) (TRINITY_DN9596), *LIPOXYGENASE* (*LOX*) (TRINITY_DN1011), *ALLENE OXIDE SYNTHASE* (*AOS*) (TRINITY_DN11235 and DN506), and *12-OXOPHYTODIENOATE REDUCTASE* (OPR) (TRINITY_DN5311) were highly expressed in the G and GY1 stages of early anther development, whereas the transcript level was decreased at advanced developmental stages (**Figure 5A**). Genes involved in the JA signal transduction pathway include *JA ZIM-DOMAIN PROTEIN* (*JAZ*) (TRINITY_DN41207 and DN14905), *MYB21* (TRINITY_DN137956), and *MYC2* (TRINITY_DN51435 and DN10382). The transcript levels of JAZs were high in the G and GY1 stages and decreased after the GY2 stage, whereas the transcript levels of MYB21 and MYC2 were high in the GY2 and Y stages during advanced anther development (**Figure 5A**). These results indicated that JA plays important roles in the anther development of lily.

### Expression of DEGs associated with phenylpropanoid biosynthesis

In the KEGG enrichment analysis of DEGs detected in the GY1, GY2, and Y stages, the second most enriched pathway was phenylpropanoid biosynthesis (**Figure 4B**). Phenylalanine metabolism is an important secondary metabolic pathway in plants, leading to the synthesis of lignin, flavonoids, and lignans (Dong and Lin, 2021). The genes participating in lignin synthesis are *PHENYLALANINE LYASE* (*PAL*) (TRINITY_DN10251 and DN22197), *COUMARIC ACID COENZYME A LIGASE* (*4CL*) (TRINITY_DN12979, DN1021, and DN3824), *CAFFEIC ACID-O-METHYLTRANSFERASE* (*COMT*) (TRINITY_DN2741), and *CAFFEOYL-COA METHYLTRANSFERASE* (*CCoAOMT*) (TRINITY_DN9630), *CINNAMOYL-COA REDUCTASE* (*CCR*) (TRINITY_DN70213), and *CINNAMYL ALCOHOL DEHYDROGENASE* (*CAD*) (TRINITY_DN245). The transcript levels of *PAL*, *4CL*, *CCoAOMT*, and *CCR* were high at the GY1 and GY2 stages, and those of *COMT* and *CAD* were high at the GY2 and Y stages (**Figure 5B**). These gene expression patterns were consistent with the timing of secondary thickening of the inner cell walls of the anther (**Figure 2**).

### Expression of DEGs associated with the pectin catabolic process

The GO enrichment analysis showed that many DEGs were enriched in the pectin catabolic process (**Figure 4A**). Pectin degradation is involved in the degradation of the septum and formation of the stomium (Wilson et al., 2011). Several groups of enzymes are involved in pectin degradation, such as polygalacturonases (PGs), pectate lyases (PLs), pectin methylesterase (PME), and pectin methylesterase inhibitor (PMEI). PME demethylates pectin and PMEI modulates PME demethylation activity, which jointly regulate the methyl level of pectin. Among the DEGs annotated with the pectin catabolic process, the transcript level of numerous genes, such as *PG* (TRINITY_DN4278 and DN13255), *PL* (TRINITY_DN6468, DN37093, and DN31526), *PME* (TRINITY_DN4610, DN157185, and DN113358), and *PMEI* (TRINITY_DN28765 and DN82103), was increased at advanced anther development (GY2, Y, and P) (**Figure 5C**). These results were consistent with the microscopic observation of septum breakdown between the GY2 and Y stages, which also coincided with stomium formation (**Figure 2**).

### Validation of DEGs by qRT-PCR analysis

**Figure 6:**
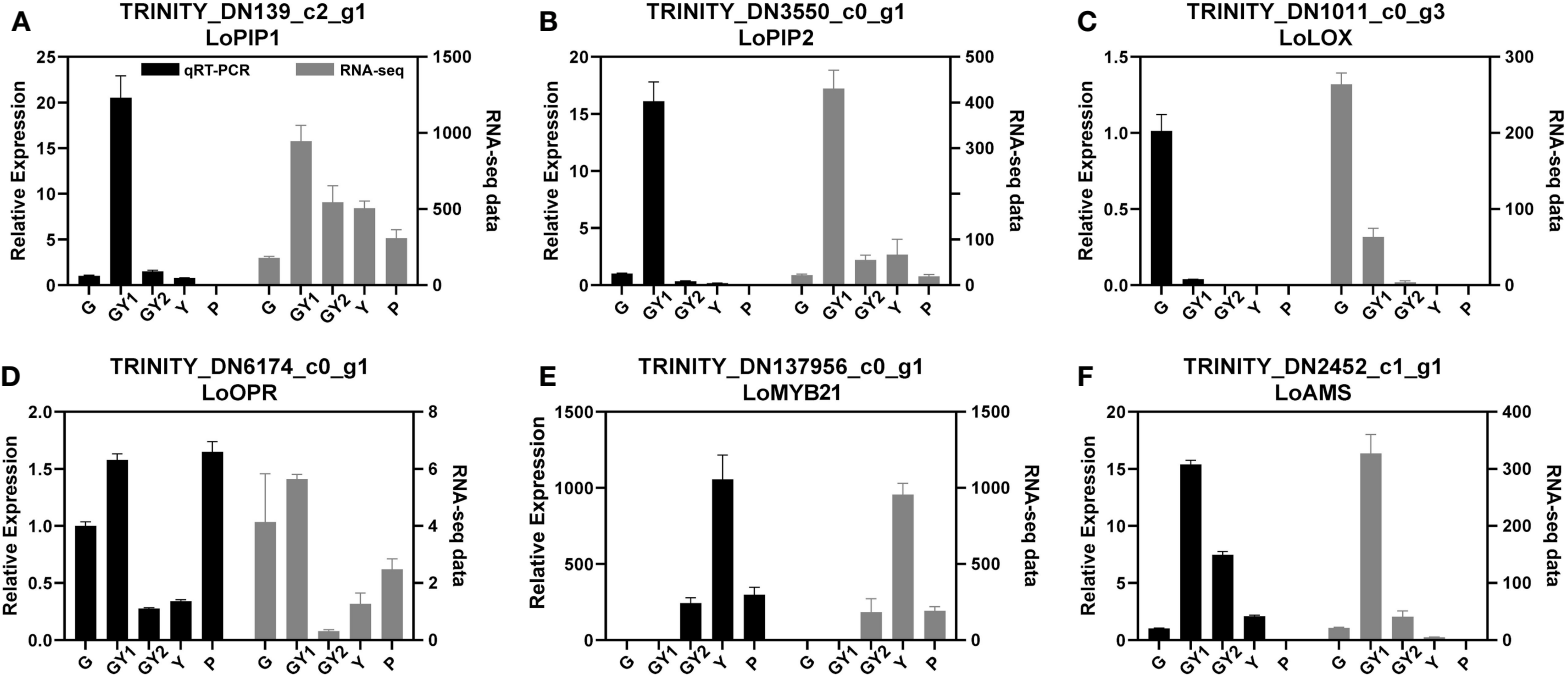
Relative expression of genes selected for validation of RNA-sequencing results by quantitative real-time PCR analysis. The genes comprised aquaporin-, jasmonic acid-, and transcription factor-related genes. The *18S* rRNA gene was used as an internal control. Error bars represent the SD. Three biological replicates were analyzed.

Three DEGs that participate in the JA metabolism and signaling pathways were chosen to validate the RNA-seq results by means of qRT-PCR analysis. The expression levels of *LoLOX* (TRINITY_DN1011), *LoOPR* (TRINITY_DN6174), and *LoMYB21* (TRINITY_DN137956) in the qRT-PCR assays were consistent with the transcriptome data. *LoLOX* and *LoOPR* were highly expressed in the G and GY1 stages, whereas *LoMYB21* was highly expressed in the Y stage, which confirmed that JA-related genes may play roles in all stages of anther development (**Figures 6C–E**).

Several genes have been reported to regulate anther development, such as *LoPIP2* (Tong et al., 2013) and *LoAMS* (Sui et al., 2020). The transcription of these genes was changed during anther development. Therefore, these genes were also selected to validate the RNA-seq results. Two aquaporin genes, *LoPIP1* (TRINITY_DN139) and *LoPIP2* (TRINITY_DN3550), were detected in the transcriptome data. The qRT-PCR results were basically consistent with the trends of the RNA-seq data, although the results for *LoPIP1* showed a slight difference between the qRT-PCR and RNA-seq analyses (**Figures 6A, B**). The gene *LoAMS* is a MYC-type transcription factor that is reported to function in anther development. The expression level of *LoAMS* was verified to be higher at GY1 and GY2 than in the other stages by qRT-PCR (**Figure 6F**).

These results indicated that the RNA-seq data were reliable and could be used for future research.

### Silencing of *LoMYB21* and *LoAMS* affected the anther development

**Figure 7:**
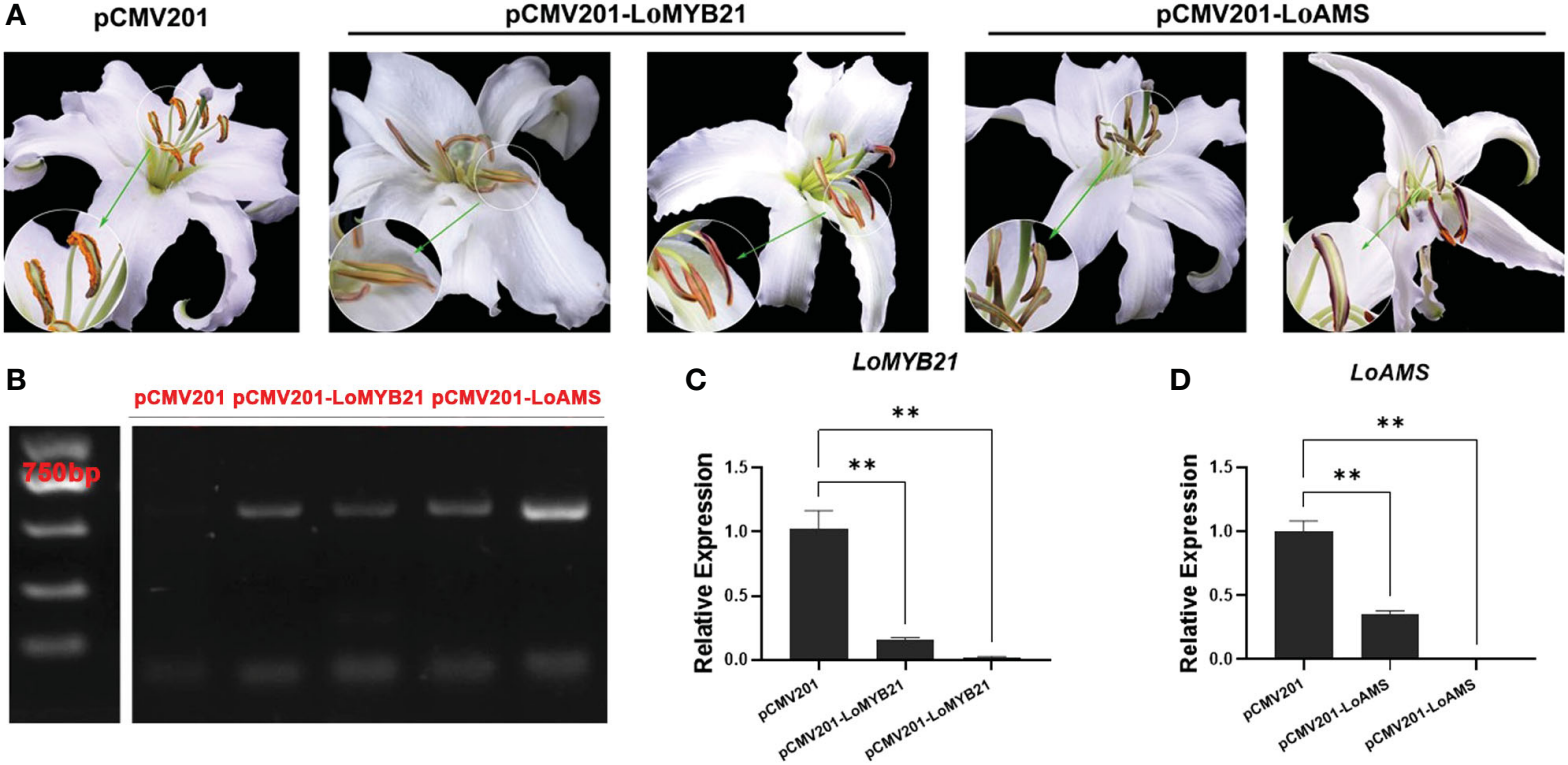
Virus-induced silencing of *LoMYB* and *LoAMS* in *Lilium* Oriental Hybrid ‘Siberia’. **(A)** Anther phenotypes of *LoMYB*-silenced and *LoAMS*-silenced plants. **(B)** Electrophoresis gel plot of the gene sequence encoding the CP protein in the cucumber mosaic virus control plants (transformed with the pCMV201 vector), *LoMYB21*-silenced plants, and *LoAMS*-silenced plants. **(C, D)** Quantitative real-time PCR analysis of *LoMYB21* and *LoAMS* expressions in the anthers of *LoMYB21*-silenced and *LoAMS*-silenced plants.

Given that the expression of *LoMYB21* and *LoAMS* changed during the development of lily anthers, the two genes were chosen for virus-induced gene silencing using a CMV system. The dehiscent standard is the state of anther when the flower opens fully. The anthers of the control (transformed with the pCMV201 vector) dehisced and released the pollen when the flower open fully, whereas the anthers of both *LoAMS*-silenced plant and *LoMYB21*-silenced plants did not dehisce or dehisced only in the apical portion of the anther at the same flower open condition. Thus, anther dehiscence was delayed in the gene-silenced plants without affecting the development of other floral organs (**Figure 7A**). Expression of the *CP* gene encoding the coat protein of the CMV virus was also detected in the gene-silenced plants, indicating that CMV infected and functioned in the silenced plants (**Figure 7B**). The expression levels of *LoMYB21* and *LoAMS* were significantly decreased in the gene-silenced plants (**Figures 7C, D**). These results indicated that *LoMYB21* and *LoAMS* may be involved in anther dehiscence and also validated the RNA seq results.

## Discussion

Lily is an important ornamental and cut flower crop worldwide; however, staining caused by the large anthers which produce the amount of pollen can be problematic. To address this issue by molecular breeding requires elucidation of the mechanisms involved in anther development and identification of the key genes that regulate pollen abortion or anther dehiscence. In recent years, several genes, such as *LoPIPI2* (Tong et al., 2013), *LoMYB80* (Sui et al., 2015), *LoAMS* (Sui et al., 2020), and *LoMYB33* (Liu et al., 2021), have been cloned and indicated to regulate different stages of anther development. Nevertheless, the delimitation of developmental stages and roles of additional genes in the regulatory mechanism require further study.

### Anther developmental stages in lily

Anther development in the model plant *Arabidopsis* has been divided into 14 stages from differentiation of the anther primordium to pollen release (Sanders et al., 1999). This classification provides a convenient framework for research on the anther development of *Arabidopsis*. In lilies, anther development has been categorized into four stages based on the anther color in a study of *LoPIP2*, which changed sequentially from white (early development) to green, yellow, and purple (maturity) (Tong et al., 2013). In addition, in research on *LoMYB80*, the development of the tapetum and microspores/pollen has been observed by sectioning paraffin-embedded floral buds of different lengths (Sui et al., 2015).

The change in anther color is a gradual process, an accurate definition of the color is difficult to provide, and it is difficult to directly observe during lily development. The length of the flower buds may partially reflect anther growth in lily because the anther length and the flower bud length showed a distinct linear relationship (**Figure 1**). Therefore, it is reasonable to use the combination of bud length, anther length, and anther color as standard criteria for delimitation of anther developmental stages. According to the morphological and anatomical results, the development of lily anther is categorized into five stages: green (G), green-to-yellow 1 (GY1), green-to-yellow 2 (GY2), yellow (Y), and purple (P) stages. The G stage shows that the flower bud length is below 4 cm, the anther color is green, and it has a typical “butterfly-shape” transverse section, which correspond to the early stage with the stamen primordia, archesporial initiation, and pollen mother cell formation (Goldberg et al., 1993; Scott et al., 2004; Tong et al., 2013; Sui et al., 2015). The GY1 stage shows that the flower bud length is between 4 and 5 cm and the anther side is turning yellow gradually and not uniformly, which correspond to the first stage of tapetum development and pollen mother cell meiosis (Goldberg et al., 1993; Sui et al., 2015). The GY2 stage shows that the flower bud length is between 6 and 7 cm and the anther side is turning yellow gradually and not uniformly, corresponding to endothecium expansion, tapetum programmed cell death, and microspore maturation (Goldberg et al., 1993; Sui et al., 2015). The Y stage shows that the flower bud length is between 8 and 10 cm and with yellow anthers, which corresponds to endothecium secondary thickening and septum degradation (Goldberg et al., 1993; Wilson et al., 2011). Finally, the P stage has the bud length at over 10 cm and with purple anthers.

This proposed standard classification of anther developmental stages in lily, based on the bud length and the length and color of the anthers, is in accordance with the anatomical observation of the paraffin sections. The classification would be suitable for future studies on the anther development of lily, has strong practicability in further research and applications, and enables approximate judgment of the developmental period of the anther without destruction of the flower bud.

### Differentially expressed genes at different stages of anther development

Anthers at five developmental stages from lily Oriental Hybrid ‘Siberia’ were collected for RNA-seq analysis. Transcriptome studies during anther development of lily are scarce because of the large genome and genetic diversity of hybrids. A total of 268.92 Gb clean data and 81,287 unigenes and 117,458 transcripts were generated from the RNA-seq data, which are similar to previously reported data using flowers or seedlings of ‘Siberia’ (Hu et al., 2017; Shi et al., 2018; Zhou et al., 2022). A large amount of annotation information was obtained from six public bioinformatic databases, and 17.92% of the sequences showed highest similarity to sequences from *Elaeis guineensis*. In addition, the PCA results showed that the biological reproducibility of the RNA-seq data was good (**Figure 3**). The present results indicated that the transcriptome profile changes during the different stages of anther development will provide useful information for future research on anther development in lily. The GO enrichment analysis suggested that the detected DEGs were involved in the pectin catabolic process, steroid metabolic process, antibiotic catabolic process, drug catabolic process, regulation of hormone levels, and hydrogen peroxide catabolic process (**Figure 4A**). The KEGG analysis revealed that the plant hormone signal transduction, phenylpropanoid biosynthesis, starch and sucrose metabolism, and flavonoid biosynthesis pathways were enriched among the DEGs (**Figure 4B**). These results indicated that the regulatory mechanism associated with anther development was highly complex and involved in the integration of numerous signaling and biosynthetic pathways.

### JA synthesis and signaling pathways are involved in anther development

Previous studies have reported that abnormal contents or signaling of JA, GAs, and auxin may lead to male sterility through the regulation of anther dehiscence and pollen maturation (Murray et al., 2003; Cecchetti et al., 2008; Cheng et al., 2009; Mutasa-Gottgens and Hedden, 2009; Song et al., 2013; Cecchetti et al., 2017; Song et al., 2018). Among the DEGs involved in hormone-related pathways, DEGs associated with auxin and GAs were the most abundant (**Supplementary Figure S7**). Given that GAs and auxin act indirectly through JA to regulate anther dehiscence and pollen maturation (Cheng et al., 2009; Song et al., 2013), the JA-related DEGs were analyzed (**Figure 5A**). The expression levels of JA biosynthesis genes, such as *PLA*, *LOX*, *AOS*, and *OPR*, were high in the early stages of anther development (G, GY1, and GY2). The JAZ genes, as inhibitors of JA signaling, were highly expressed in the early developmental stages, whereas the expression levels of *MYB21* and *MYC2* were high at the GY2 and Y stages (**Figure 5A**). The expression of these genes was verified by qRT-PCR analysis (**Figure 6A**). In addition, anther dehiscence in *LoMYB21*-silenced plants was delayed compared with the control plants (**Figure 7A**). These results were consistent with previous studies on *Arabidopsis* mutants that demonstrate how various genes associated with the JA biosynthesis and signaling pathways affect anther dehiscence, such as *dad1* and *opr3* mutants, in which male sterility can be rescued by exogenous JA application (Stintzi and Browse, 2000; Ishiguro et al., 2001; Cheng et al., 2009). These results suggest that genes associated with the JA metabolism and signaling pathways have roles in anther development in lily.

### Roles of phenylpropanoid biosynthesis in anther dehiscence

One product of phenylpropanoid biosynthesis is lignin, the accumulation of which contributes to secondary cell wall thickening (Dong and Lin, 2021). Among the DEGs involved in phenylpropanoid biosynthesis, *PAL*, *4CL*, *COMT*, *CCR*, and *CAD* were highly expressed at the GY1 and GY2 stages, and COMT and CAD were also highly expressed at the Y stage (**Figure 5B**). These results were consistent with the study of *Arabidopsis* mutants defective in phenylpropanoid metabolism. The phenylpropanoid biosynthesis triple mutant *cadc cadd ccr1* (*ccc*), in which two *CAD* genes (*CADc* and *CADd*) and one *CCR* gene were disrupted, showed reduced lignification in the anther endothecium, resulting in the failure of anther dehiscence and pollen release (Thévenin et al., 2011). Studies on *Arabidopsis nst1 nst2* and *myb26* mutants have shown that secondary wall thickening, specifically in the endothecium, is necessary for anther dehiscence (Mitsuda et al., 2005; Yang et al., 2007b; Yang et al., 2017).

### Pectin catabolic process affects the advanced anther development

The pectin catabolic process plays a crucial role in anther development degradation of the septum and formation of the stomium (Keijzer, 1987). Key enzymes mediate the removal of methyl-ester groups from unbranched homogalacturonan, which is one type of pectic polysaccharide. Pectin methyl-esterases were highly expressed at the advanced stages of anther development (GY2, Y, and P), during which septum degradation occurs (**Figure 5C**). Several PGs and PLs showed a similar expression pattern, which cleaved homogalacturonan during pectin metabolism (**Figure 5C**). Previous studies have shown that the pectinase-related PG genes mainly play a role in the process of cell separation, which is essential for abscission and anther dehiscence (Yang et al., 2013; Ye et al., 2020). In *Arabidopsis*, ADPG1, ADPG2, and QUARTET2 are PG genes that contribute to anther dehiscence (Ogawa et al., 2009). In the tomato (*Solanum lycopersicum*) *ps-2* mutant, non-dehiscence of the anthers is caused by a single nucleotide mutation in a novel tomato PG gene (Gorguet et al., 2009). Several PG or PL genes perform functions in pollen development in *Brassica campestris*, including *BcMF6*, *BcMF9*, *BcMF10*, *BcMF16*, and *BcMF17* (Zhang et al., 2008; Zhang et al., 2011; Zhang et al., 2012; Jiang et al., 2014a; Jiang et al., 2014b). These results suggest that the pectin catabolic process plays an important role in anther development in lily.

### Water potential participates in anther dehiscence

Previous research has shown that water status is a critical factor in anther dehiscence (Keijzer, 1987; Bots et al., 2005a; Bots et al., 2005b; Tong et al., 2013). Therefore, aquaporins were analyzed in the present RNA-seq data (**Figure 6**). Two aquaporins, PIP1 and PIP2, were highly expressed at the GY1 stage and also were expressed in the other stages of anther development (**Figure 6**). This finding was consistent with a previous report that LoPIP1 and LoPIP2 show a stable expression throughout all stages of anther development in lily (Tong et al., 2013). In tobacco, two aquaporins are specifically expressed in the anther and stylar tissues, and PIP2 is required for efficient anther dehydration before dehiscence (Bots et al., 2005a; Bots et al., 2005b). In lily, only silencing of *LoPIP2* inhibited anther dehiscence (Tong et al., 2013). The RNA-seq data reveals that the polysaccharide catabolic process and starch and sucrose metabolism were enriched among the DEGs, which could affect the water potential in the stomium (**Figure 4**). These results suggested that dehydration of the cell wall associated with the dehiscent region is necessary for anther dehiscence.

## Conclusion

The analysis of the transcriptome during anther development in lily revealed that 268.92 Gb clean reads were generated and 81,287 unigenes were annotated. The transcriptome data provides valuable genetic resources for gene isolation in future anther-related research on lily. The present analyses indicated that JA, lignin, and pectin metabolism play important roles in anther development and that *LoMYB21* regulates anther dehiscence without affecting the development of other floral organs in lily. Future studies to confirm the molecular functions of these genes will provide novel data for understanding the regulatory mechanism of anther development in lily and other plants.

## Data availability statement

The datasets presented in this study are deposited in the National Center for Biotechnology Information BioProject, accession: PRJNA923113.

## Author contributions

JH, TD, and LW conceived the research and designed the experiments. LW, TD, RW, and XY performed the experiments and analyzed the data. LW and TD wrote the manuscript. WJ, MY, XZ, and JH revised and edited the manuscript. All authors contributed to the article and approved the submitted version.
