# Supplementary material for: Transcriptomic analysis reveals candidate genes associated with anther development in Lilium Oriental Hybrid ‘Siberia’
Source: Front Plant Sci. 2023 Feb 8;14:1128911. doi: 10.3389/fpls.2023.1128911 (PMC9945121; doi:10.3389/fpls.2023.1128911)
Supplement: Supplementary file 1 [file DataSheet_1.doc]

Supplementary Material

# Transcriptomic analysis reveals candidate genes associated with anther development process in lilium oriental 'Siberia'

Tingting Dong ^1#^, Lixuan Wang ^1#^, Rui Wang ^1^, Xi Yang ^1^, Wenjie Jia ^1,2^, Mingfang Yi ^1^, Xiaofeng Zhou ^1^, Junna He ^1*^

*** Correspondence:** Junna He: [hejunna@cau.edu.cn](mailto:hejunna@cau.edu.cn)


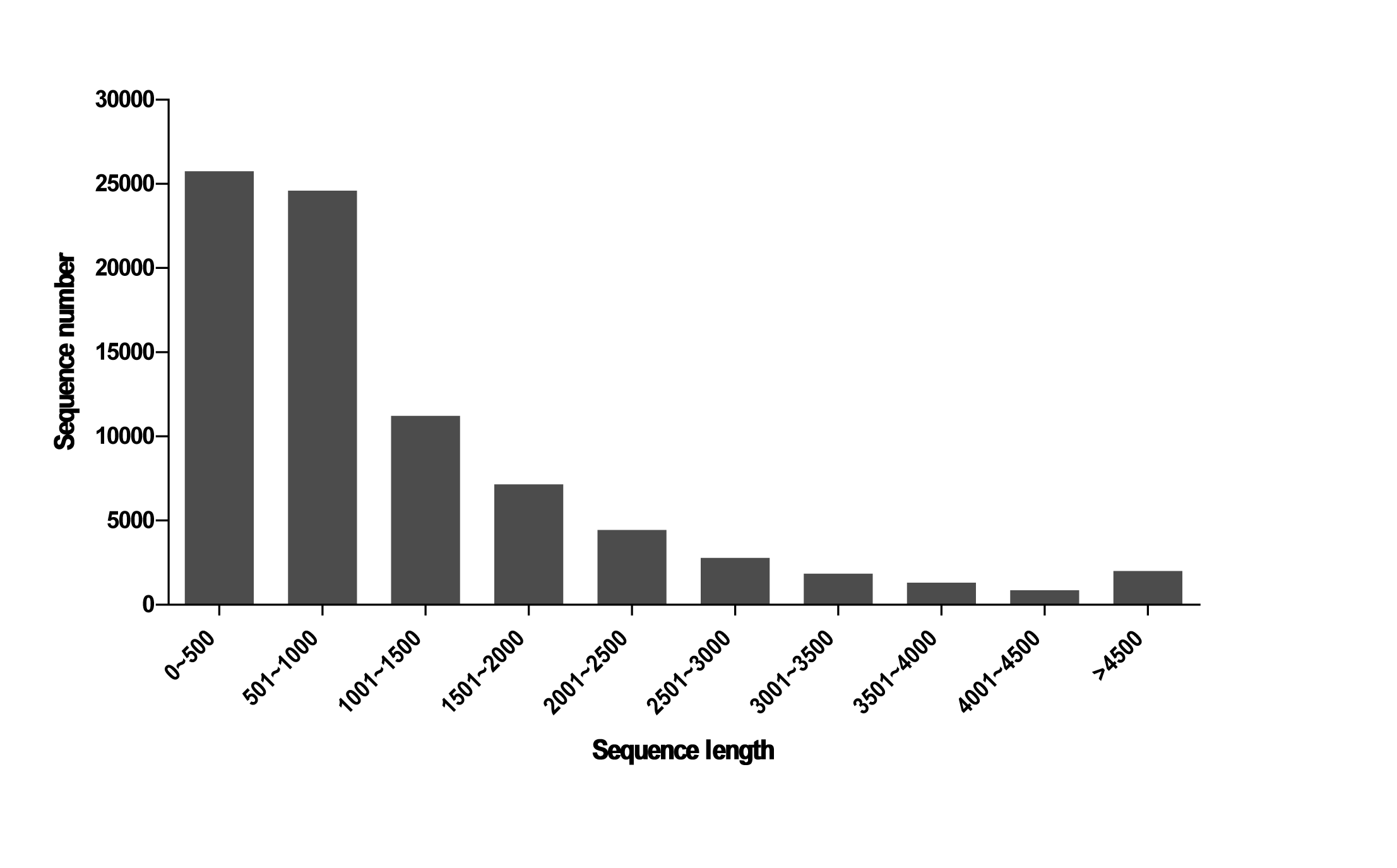


**Supplementary Figure 1.** Length distribution of transcript sequences.


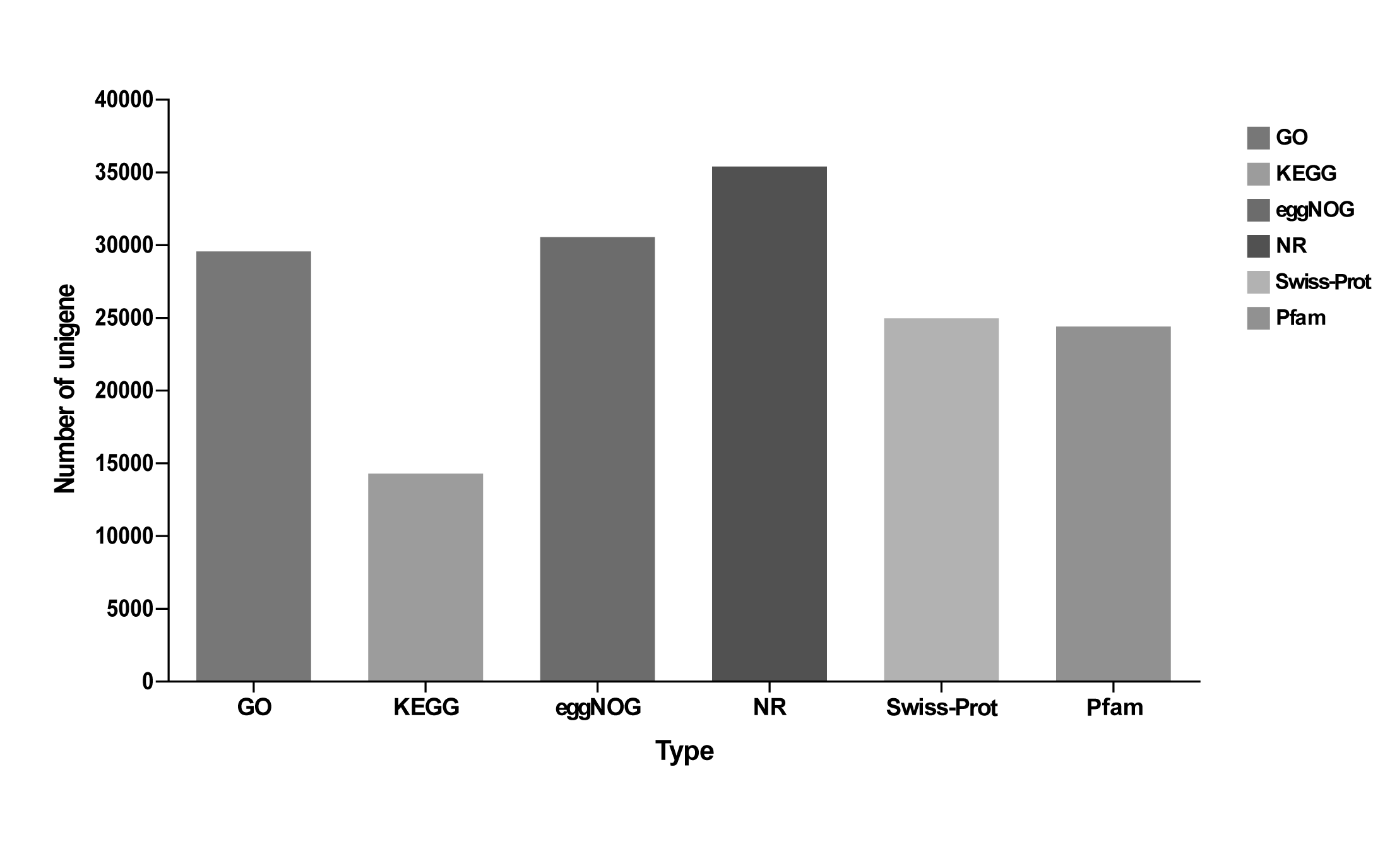


**Supplementary Figure 2.** Unigene functions were annotated by six databases.


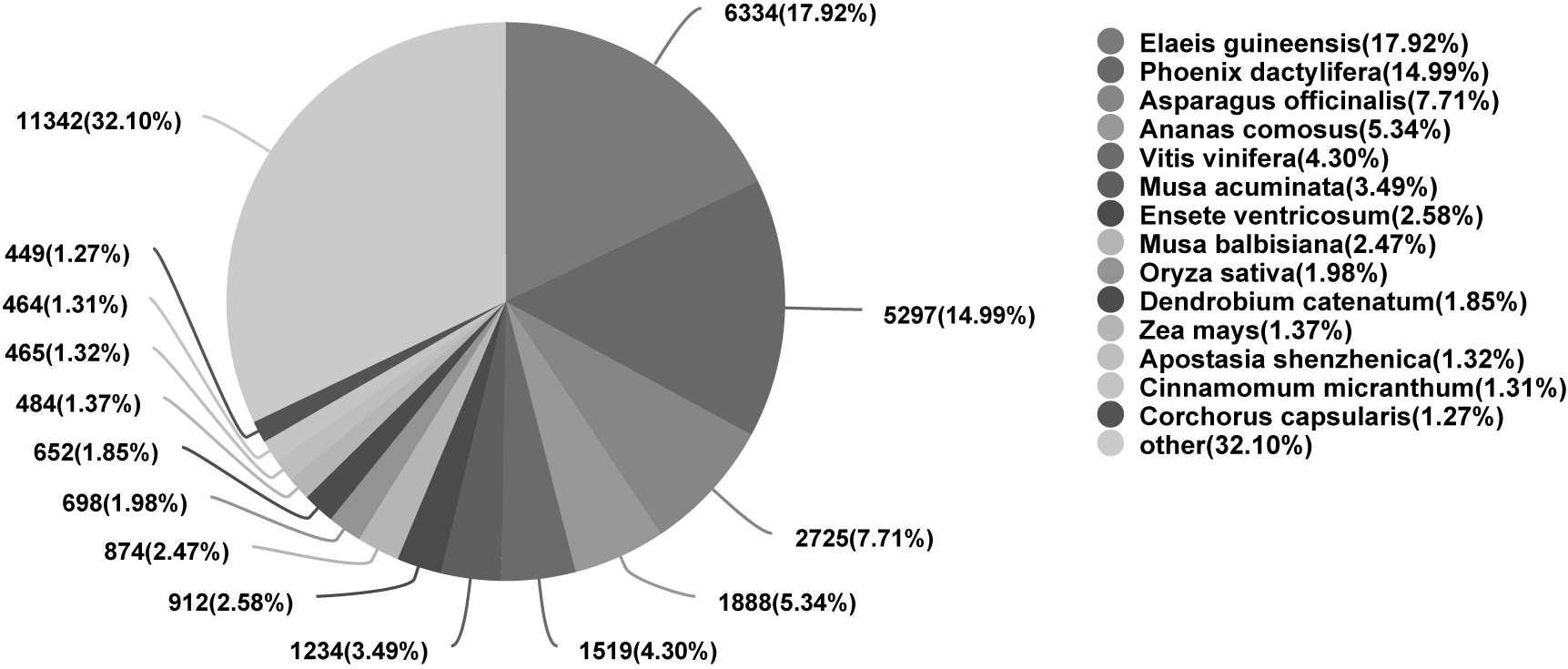


**Supplementary Figure 3.** Species distribution by NR annotation.


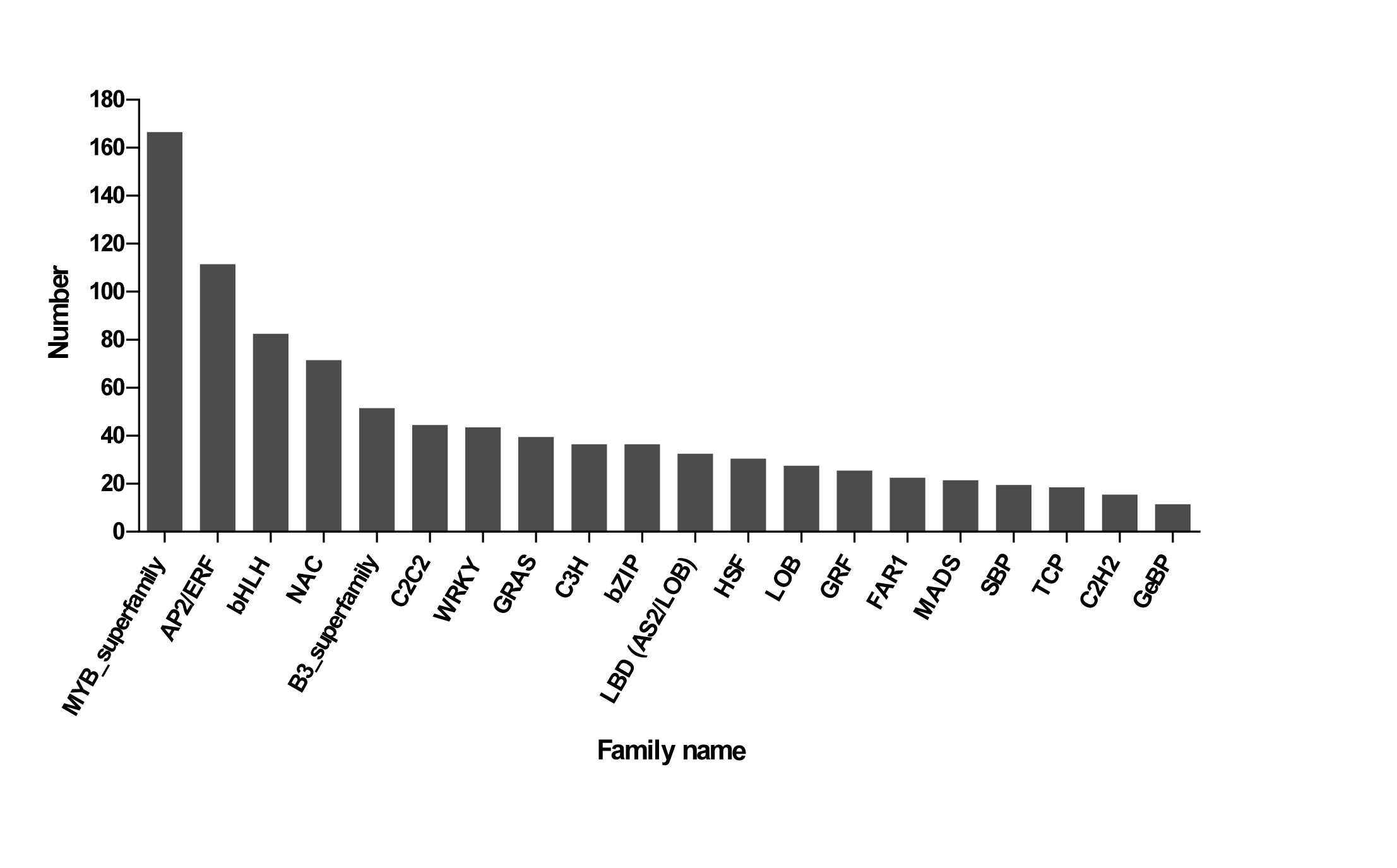


**Supplementary Figure 4.** Transcription factor analysis of the assembled sequence.


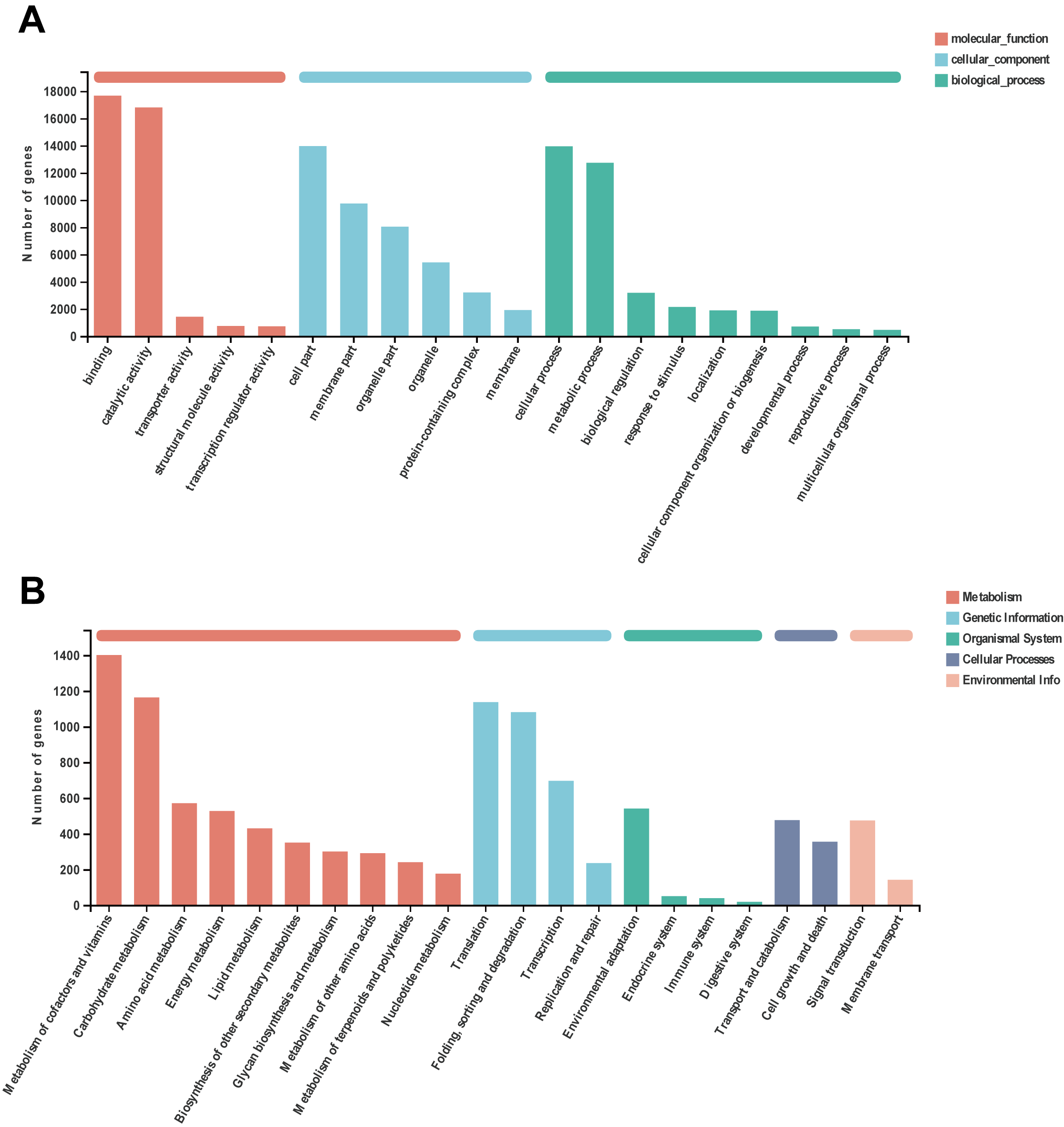


**Supplementary Figure 5.** Unigene functions were annotated by GO (Gene Ontology) and KEGG (Kyoto Encyclopedia of Genes and Genomes) databases.


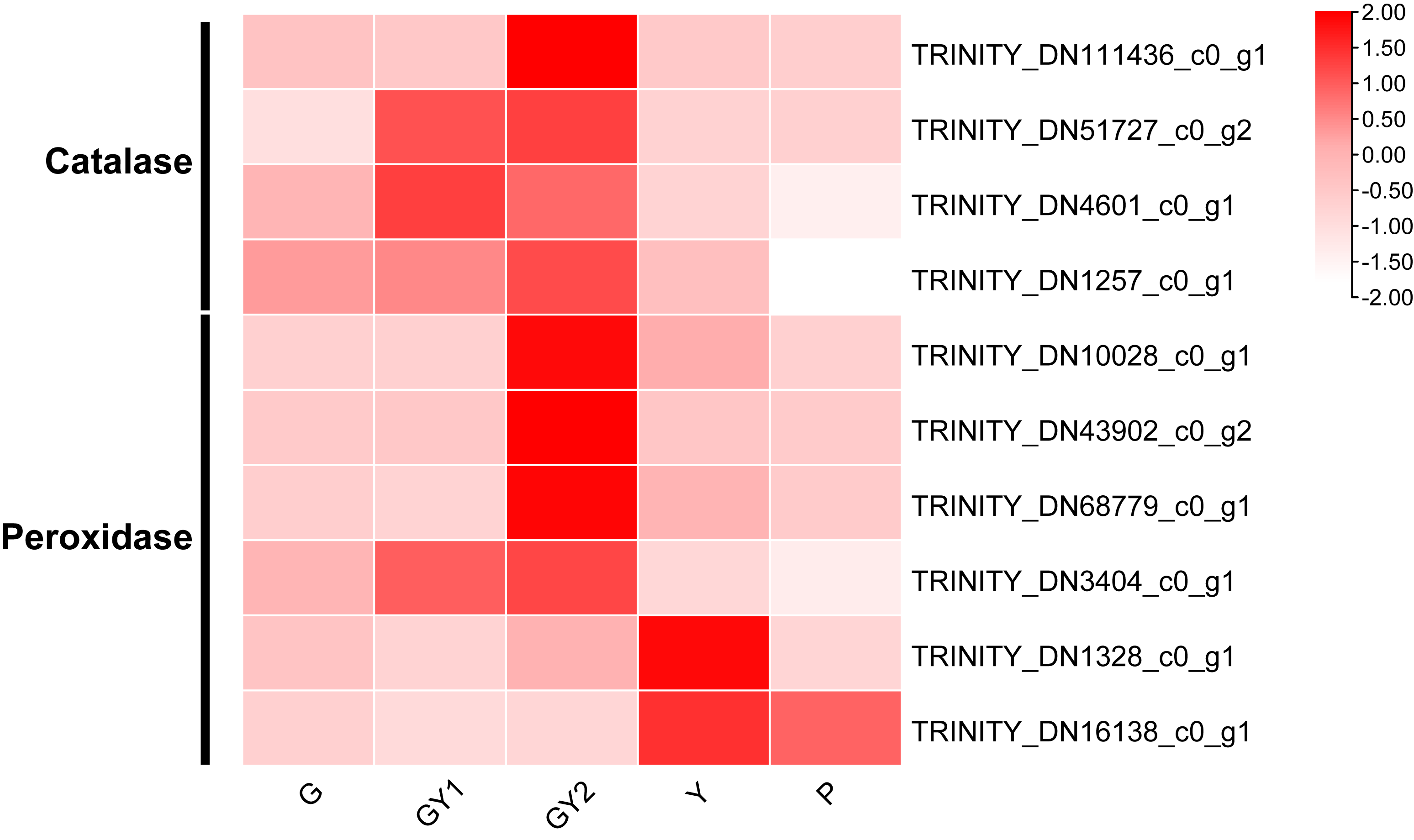


**Supplementary Figure 6.** Heat map of expression of DEGs related with ROS metabolic enzyme. The heat map shows the expression of catalase and peroxidase in the ROS pathway at different stages of anther development. G: green stage; GY1: green to yellow stage 1; GY2: green to yellow stage 2; Y: yellow stage; P: purple stage.


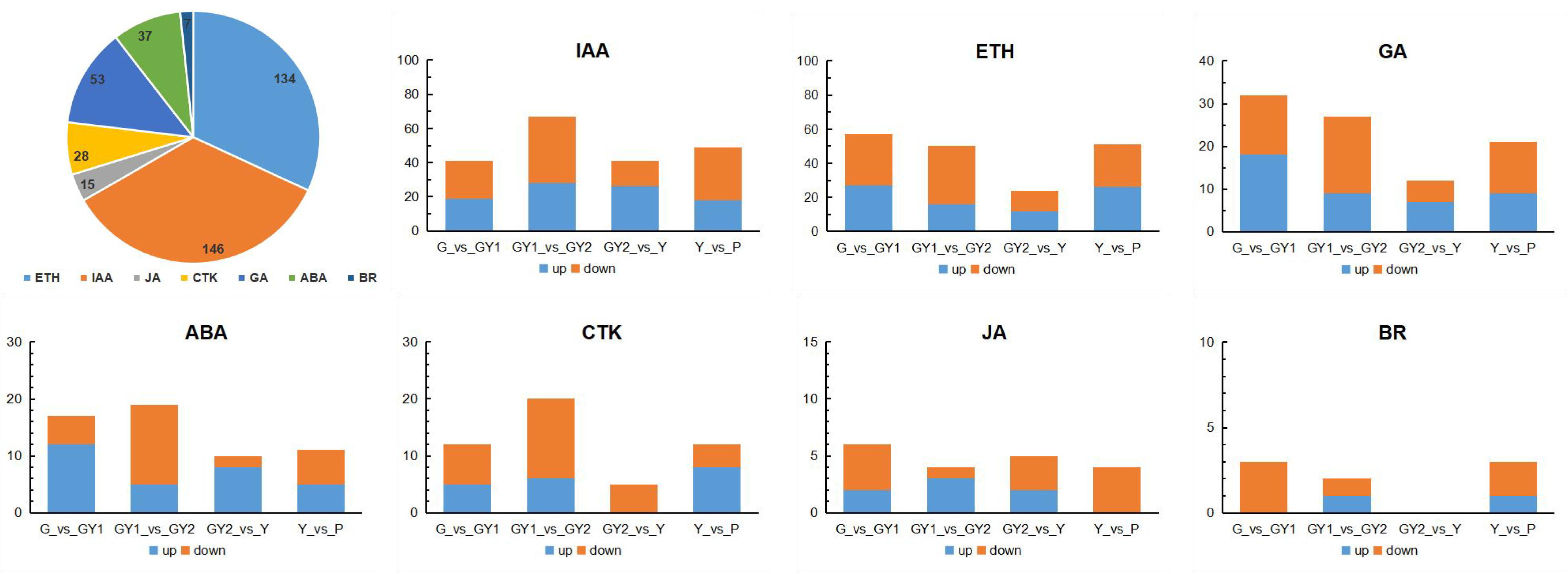


**Supplementary Figure 7.** Analysis of DEGs related with plant hormones. The figure includes the number of DEGs for hormones, such as auxin (IAA), ethylene (ETH), gibberellin (GA), abscisic acid (ABA), cytokinin (CTK), jasmonic acid (JA), and brassinosteroids (BR), in comparison of different developmental groups.

**Supplementary Table1.** Quality control data statistics

| **Sample** | **Clean reads** | **Clean bases** | **Q30(%)** |
| --- | --- | --- | --- |
| G_1 | 116040446 | 17386399874 | 92.44 |
| G_2 | 130428710 | 19541118704 | 91.92 |
| G_3 | 102412286 | 15344752062 | 93.54 |
| GY1_1 | 109608638 | 16421856035 | 92.78 |
| GY1_2 | 116693622 | 17482898555 | 92.58 |
| GY1_3 | 136491784 | 20449741775 | 92.62 |
| GY2_1 | 119705972 | 17935039177 | 92.28 |
| GY2_2 | 103312808 | 15480033575 | 92.67 |
| GY2_3 | 116008464 | 17382869089 | 93.17 |
| Y_1 | 115182484 | 17259474756 | 92.77 |
| Y_2 | 103357416 | 15487975716 | 93.38 |
| Y_3 | 95014252 | 14236738828 | 92.84 |
| P_1 | 162576052 | 24354326677 | 92.62 |
| P_2 | 148701492 | 22276214012 | 92.65 |
| P_3 | 119330068 | 17876680968 | 92.65 |

**Clean reads:** count the number of sequencing data after filtering; **Clean bases:** The number of clean reads is multiplied by the length and converted to G as the unit; **Q30:** Calculate the percentage of bases with P values greater than 30 in the total bases.

**Supplementary Table2. Data analysis statistics**

| **Type** | **Unigene** | **Transcript** |
| --- | --- | --- |
| Total number | 81287 | 117458 |
| N50 length (bp) | 1791 | 1805 |
| Fragment mapped percent(%) | 71.446 | 80.705 |
| GC percent (%) | 42.78 | 43.26 |
| BUSCO score | C:79.9%[S:77.6%;D:2.3%] | C:79.9%[S:77.6%;D:2.3%] |

**Total num:** the number of assembled unigenes/transcripts; **N50:** the length of the accumulated transcript reaches half of the total length; **Fragment mapped reads:** All sample clean reads are merged and compared with the assembled unigene/transcript, and the obtained mapped reads; **GC percent:** the percentage of the total number of GC bases in the total number of bases; **Busco score:** using BUSCO to evaluate the integrity of the assembly Carry out the evaluation score, "C" stands for complete means that in the assembled sequence on the alignment, the proportion of the sequence with the desired length to the total sequence of busco, which consists of two parts S and D, "S" stands for Single-copy that is a sequence can be compared to one gene in the library; "D" means duplicate that is a sequence can be compared to multiple genes in the library .

**Supplementary Table 3** The primers of DEGs used RT-PCR

| **primer** | **sequence（5'—3'）** |
| --- | --- |
| CMV-CP-F | TGCTACCTTTAGAGTCCTGTCGCAGC |
| CMV-CP-R | AGCACTCCAGATGTGGGAATACGTTG |
| qRT-LoPIP1-F | CACTGGCATCAACCCTGCTA |
| qRT-LoPIP1-R | TGGAACCTTTGTCCTCGTCTAC |
| qRT-LoPIP2-F | TGGAACCTTTGTCCTCGTCTAC |
| qRTLoPIP2-R | AGCTCCGGTAGGATCCGAG |
| qRT-LoLOX-F | CATTCTGTCCAACCACTCGCCAG |
| qRT-LoLOX-R | GGCCTCAACAATTCATACGGCACT |
| qRT-LoOPR-F | TCCTCTACTGCCACATGACCGAAC |
| qRT-LoOPR-R | CTCATTCAGCGGTGCATTTAGCTC |
| qRT-LoAMS-F | ACAAACACTCTTGGCAATGCAACCT |
| qRT-LoAMS-R | ATGGTGTGCTGATCCTCTACAACCT |
| qRT-LoMYB21-F | GTCTGGAACACGCTAGCAAA |
| qRT-LoMYB21-R | TGTTATATTTCCCCGACGGACA |
| qRT-Lo18S-F | AGTTGGTGGAGCGATTTGTCT |
| qRT-Lo18S-R | CCTGTTATTGCCTCAAACTTCC |
